# Role of Retinoblastoma Protein Family (Rb/p105 and Rb2/p130) Expression in the Histopathological Classification of Borderline Ovarian Tumors

**DOI:** 10.3389/fmed.2020.596226

**Published:** 2020-11-11

**Authors:** Valeria Masciullo, Paola Valdivieso, Giulia Amadio, Angela Santoro, Giuseppe Angelico, Alessandro Sgambato, Silvia Boffo, Antonio Giordano, Giovanni Scambia, Gian Franco Zannoni

**Affiliations:** ^1^Unità di Ginecologia Oncologica, Dipartimento Scienze della Salute della Donna, del Bambino e di Sanità Pubblica, Fondazione Policlinico Universitario A. Gemelli IRCCS, Roma, Italy; ^2^Unità di Gineco-Patologia e Patologia Mammaria, Dipartimento Scienze della Salute della Donna, del Bambino e di Sanità Pubblica, Fondazione Policlinico Universitario A. Gemelli IRCCS, Roma, Italy; ^3^Istituto di Patologia Generale, Università Cattolica del Sacro Cuore, Roma, Italy; ^4^Department of Biology, College of Science and Technology, Sbarro Institute for Cancer Research and Molecular Medicine, Temple University, Philadelphia, PA, United States; ^5^Istituto di Clinica Ostetrica e Ginecologica, Università Cattolica del Sacro Cuore, Roma, Italy; ^6^Istituto di Anatomia Patologica, Università Cattolica del Sacro Cuore, Roma, Italy

**Keywords:** pRb/p105, pRb2/p130, diagnosis, retinoblastoma protein family, borderline ovarian tumors

## Abstract

Borderline ovarian tumors (BOT) are uncommon but not rare epithelial ovarian neoplasms, intermediate between benign and malignant categories. Emerging knowledge supports the notion that subtypes of borderline ovarian tumors comprise distinct biologic, pathogenetic, and molecular entities, precluding a single unifying concept for BOT. The identification of valuable markers for the diagnosis and classification of these tumors is in need. Among the molecular candidates, the Retinoblastoma (Rb) family members Rb/p105 and Rb2/p130 seem to play a pivotal role in ovarian cancer. In particular, Rb/p105, when in the unphosphorylated form, acts as a growth suppressor controlling cell cycle and tumor progression; whereas, the phosphorylated form activates gene transcription and cellular proliferation. While Rb/p105 is ubiquitously confined to the nuclei of cycling and quiescent cells, Rb2/p130 activity is also regulated by intracellular localization. According to this, Rb family members could represent a novel marker in diagnosis and classification risk for patients with BOT. In this study, we evaluated the expression and subcellular localization of proteins of the retinoblastoma (Rb) gene family in 65 ovarian borderline tumors. Statistically significant differences were found in nuclear and cytoplasmic expressions of Rb/p105 and Rb2/p130 according to different examined histotypes. In detail, the nuclear expression of Rb/p105 and Rb2/p130 was more frequently detected in serous (84.6%) than sero-mucinous (42.1%) and mucinous (50%) types. Conversely, the cytoplasmic expression of Rb2/p130 was not detected in serous tumors and frequently observed in mucinous subtypes (80%). Our findings suggest that Rb proteins do not play a key role in the tumor progression of serous borderline tumors since any cases showed cytoplasmic localization. By contrast, the observed higher cytoplasmic expression of Rb2/p130 in intestinal mucinous BOTs is indicative of Rb protein family involvement in the cancerogenesis pathway of mucinous ovarian tumors. Also, mucinous BOTs of intestinal-type, exhibiting low nuclear and high cytoplasmic levels of Rb2/p130 might potentially be considered a high-risk category of malignant evolution. Further studies on larger series are needed to clarify how BOTs could be stratified in different prognostic groups according to their Rb proteins immunohistochemical profile.

## Background

Borderline ovarian tumors (BOTs) represent one of the controversial topics in gynecologic pathology (1). They are a heterogeneous group of tumors that account for 10–20% of all ovarian epithelial neoplasms. The most common BOT histotypes are serous (50%) and mucinous (45%) with less common subtypes (5%) including sero-mucinous, endometrioid, clear cell, and borderline Brenner tumors (2, 3). The 97% of all stages of BOT have a good prognosis with a mean 10-years survival (4), although recurrences and malignant transformation can occur in a very small proportion of cases (5). In contrast to serous BOTs, that are rarely characterized by evolution in a low-grade serous carcinoma whereas are often associated with peritoneal implants and relapses (5), mucinous carcinoma frequently develops from benign and borderline mucinous tumors (6, 7). Similarly, sero-mucinous BOTs are often the land of endometrioid or clear cell carcinoma and usually represent a morphologic continuum in the middle of benign and malignant counterparts.

BOTs often occur in young women, however, the absence of stromal invasion warrants a better prognosis compared to ovarian carcinoma (8, 9). Nevertheless, since the standard treatment of BOTs is usually surgery, the fertility of these women may be affected (10). Identifying the genetic background for diagnosis and prognosis should avoid a radical resection and help in developing new targeted therapies, especially in younger women with a desire for childbearing. Thus, a better understanding of the clinical phenotype and pathogenesis of BOTs would contribute to their earlier detection and is essential for the development of more effective treatments.

Previous studies support the idea that the serous and mucinous BOT have distinct carcinogenic pathways. For example, the expression of p21 and MDM2 differs between mucinous and serous forms (11). In other studies, a higher rate of p53 mutation was observed in mucinous compared to serous BOTs and p21 and bcl-2 overexpression appeared specific to serous forms and different between serous benign, serous BOTs and serous carcinoma (12–14).

The retinoblastoma gene family consists of three members and their products are Rb/p105, Rb2/p130, and RbL1/p107, together known as “pocket proteins” family (15, 16). Their most important target is the E2F-family of transcription factors, which control the expression of genes that mediate G1-S transition (15, 16). The localization of these proteins into the nucleus or around the nuclear membrane has been shown at the molecular level during the different phases of the cell cycle (17, 18). In detail, during the cell progression through the S into the G2/M phases of the division cycle, pRB undergoes phosphorylation, while in the late M phase, pRB is rapidly dephosphorylated. When pRB is in the unphosphorylated form, it acts as a growth suppressor by repressing transcription of E2F. By contrast, the phosphorylated pRB status (p-pRB) leads to the activation of E2F-responsive genes and entry into the S phase. While Rb/p105 is ubiquitously confined to the nuclei of cycling and quiescent cells, Rb2/p130 activity is also regulated by intracellular localization. The phosphorylation status of Rb2/p130 itself, therefore, is important in the regulation of the cell cycle (19). The hyperphosphorylated form of pRb2/p130 is cytoplasmic and typical of cells progressing into the G1 phase (20). Alteration of Rb family members is frequently involved in gynecological cancers (21–23). We previously showed that the loss of Rb2/p130 or its cytoplasmic expression occurs in 40% of ovarian tumors and is inversely correlated with tumor grade (24). However, little is known about Rb proteins expression in borderline ovarian tumors.

In this study, we utilized immunohistochemistry to evaluate the expression pRb/p105 and pRb2/p130 family members in a large, single-institution, and series of mucinous, sero-mucinous and, serous BOTs.

## Materials and Methods

### Data Collection

Sixty-five ovarian BOTs were retrospectively collected from patients who underwent salpingo-oophorectomy for ovarian cancer in the Division of Gynecologic Oncology of the Fondazione Policlinico Universitario A.Gemelli IRCCS, Rome, Italy between 2010 and 2016; all selected patients did not receive chemotherapy or radiotherapy before surgical enucleation. All the subjects gave written informed consent before enrollment. Twenty specimens were defined as mucinous BOTs, 19 were classified as sero-mucinous, whereas 26 cases were classified as serous BOTs. Histological classification of tumors was carried out according to the WHO system, and disease staging was established according to the International Federation of Gynecologists and Obstetricians (FIGO) criteria.

### Immunohistochemistry

After surgical resection, tissues were immediately fixed in 10% formalin and then paraffin-embedded for immunohistochemical analysis. The immunostaining was performed using a streptavidin-biotin complex immunoperoxidase method (DakoCytomation). Detection for the retinoblastoma gene family members was performed using purified mouse anti-human retinoblastoma protein (Rb) monoclonal antibody (BD Pharmigen) diluted 1:50 and mouse monoclonal antibody Rb2 p130 (clone 130-P215; Novus Biologicals, Inc.) diluted 1:25.

Paraffin blocks of each specimen were sectioned at 3 μm, mounted on a slide, and dried overnight at 37°C. All sections were dewaxed in xylene and dehydrated in descending graded alcohols to Phosphate-Buffered Saline (PBS; pH 7.4).

Antigen retrieval was performed by microwaves in a 10 mM citrate buffer (pH 6), at 750 W for 10 min (two cycles of 5 min each), followed by cooling at room temperature for at least 20 min before incubation with the antibodies. Sections were treated with 0.4% H_2_O_2_ methanol solution (15 min at room temperature to inhibit endogenous peroxidase activity), quickly rinsed in water, and then in PBS.

Sections were then placed in a humidified chamber and incubated with primary antibody at room temperature for 40 min. The sections were then washed in PBS (two times for 5 min each).

Antigen detection was carried out by exposure to a biotinylated universal secondary antibody for 10 min followed by a streptavidin-peroxidase complex working solution for 10 min.

After another PBS wash, the antigen-antibody complex was visualized by staining with the chromogen 3,3′-diaminobenzidine/ tetrachloride solution (DAB, Vector) for 5 min. The sections were rinsed in deionized water; cell nuclei were counterstained with hematoxylin and dehydrated in graded alcohols followed by xylene.

Specimens of human colon cancer, follicular cyst, and fallopian tube served as positive controls for pRb and pRb2, respectively. For negative control, slides were simultaneously incubated with PBS in the absence of the primary antibody. The results were independently reviewed by three experienced pathologists (GFZ, GA, and AS), who were blinded to the clinical outcome at the time of evaluation. Discrepancies in the evaluation (<10% of cases) were resolved by re-observation of the cases using a multi-headed microscope.

For each sample, at least 20 high-power fields were randomly chosen and ~2,000 cells were counted. Quantitative scoring of protein expression was based on the percentage of positive cells as follows: negative (0%); lower positive (1–30%) or upper positive (>30%) cells). Cases showing a value more than the median (30%) of immunoreactive neoplastic cells were considered as evidence of upper positivity. In this way a cut-off of 30% was considered statistically significant and, therefore, functionally operative.

### Statistical Analysis

The associations between Rb proteins staining and other clinic-pathological parameters were analyzed using contingency table methods and tested for significance using the Fisher's exact χ2 test. All calculations were performed using the Statistical Package for Social Science (SPSS 17.0 software, Chicago, IL) and the result was considered statistically significant when the *P*-value was ≤ 0.05.

## Results

### Clinic-Pathological Features

The series included 20 mucinous, 19 sero-mucinous, and 26 serous BOTs. The mean and median age of the patients were 44.7 and 44 years (range 20–72), respectively. All mucinous and sero-mucinous selected tumors (39) were stage 1, without evidence of implants or recurrences, whereas serous tumors included stage 1 (*n* = 20), stage 2 (*n* = 1), and stage 3 (*n* = 5) cases and follow-up data were available for all 65 patients. Five serous BOTs were classified as BOTs with microinvasive foci. One mucinous BOT showed foci of intraepithelial carcinoma and an area of malignant invasive mucinous carcinoma with an expansive pattern of growth.

Fifty-nine cases were limited to the ovary, without peritoneal implants and six cases (all serous BOTs) were associated with peritoneal implants (only one of invasive type). Sixty-one cases did not relapse; the remaining four developed peritoneal recurrences. By the time this study was undertaken, no patients had died of the disease.

The clinic-pathologic characteristics of patients are summarized in Table 1. The expression levels of Rb/p105 and Rb2/p130 were determined by immunohistochemistry.

**Table 1:** Clinical data of the patients with BOTs.

	**N^**°**^ (% of Cases)**
**Histologic types**
Serous subtype^*^	26 (40.0)
Sero-Mucinous subtype	19 (29.2)
Mucinous subtype^**^	20 (30.8)
**Stage**	
1	59 (90.8)
2	1 (1.5)
3	5 (7.7)
**Implants**	
Not Implants	59 (90.8)
Implants without invasion	5 (7.7)
Implants with invasion	1 (1.5)
**Recurrences**	
No	61 (93.8)
Yes	4 (6.2)
**Clinical outcome**	
Dead	0
Alive	65 (100)

* *5 serous BOTs showed micro-invasive foci*.

** *1 mucinous BOT showed foci of intraepithelial carcinoma and an area of invasive mucinous carcinoma with expansive pattern of growth*.

### Correlation of Rb/p105 With Clinic-Pathological Parameters in BOTs

The expression of Rb/p105 according to clinic-pathological parameters is shown in Table 2.

**Table 2:** Nuclear Distribution of Rb/p105-negative and Rb/p105-positive cases according to tumoral characteristics.

**Rb/p105 Nuclear**	**Total**	**Rb/p105 negative (0%)**	**Rb/p105 positive (1–30%)**	**Rb/p105 positive (>30%)**	***P***
	**N^**°**^**	**N^**°**^ (%)**	**N^**°**^ (%)**	**N^**°**^ (%)**	
**Histologic types**	65	25 (38.4)	33 (50.8)	7 (10.8)	
Serous subtype	26	4 (15)	22 (84.6)	0 (15.4.)	<0.0001
Sero-Mucinous subtype	19	11 (57.9)	6 (31.6)	2 (10.5)	
Mucinous subtype	20	10 (50.0)	5 (25.0)	5 (25.0)	
**Stage**					0.04
1	59	25 (42.3)	27 (45.8)	7 (11.9)	
2+3	6	0	6 (100)	0	
**Implants**					NS
Absent	59	24 (41.4)	27 (46.5)	7 (12.1)	
Present	6	1 (14.3)	6 (85.7)	(0)	
**Recurrences**					NS
Absent	61	24 (39.4)	31 (50.8)	6 (9.8)	
Present	4	1 (25.0)	2 (50.0)	1 (25.0)	

*Numbers in parentheses represent the percentage of specimens achieving that particular score. NS, not significant*.

**Figure 1:**
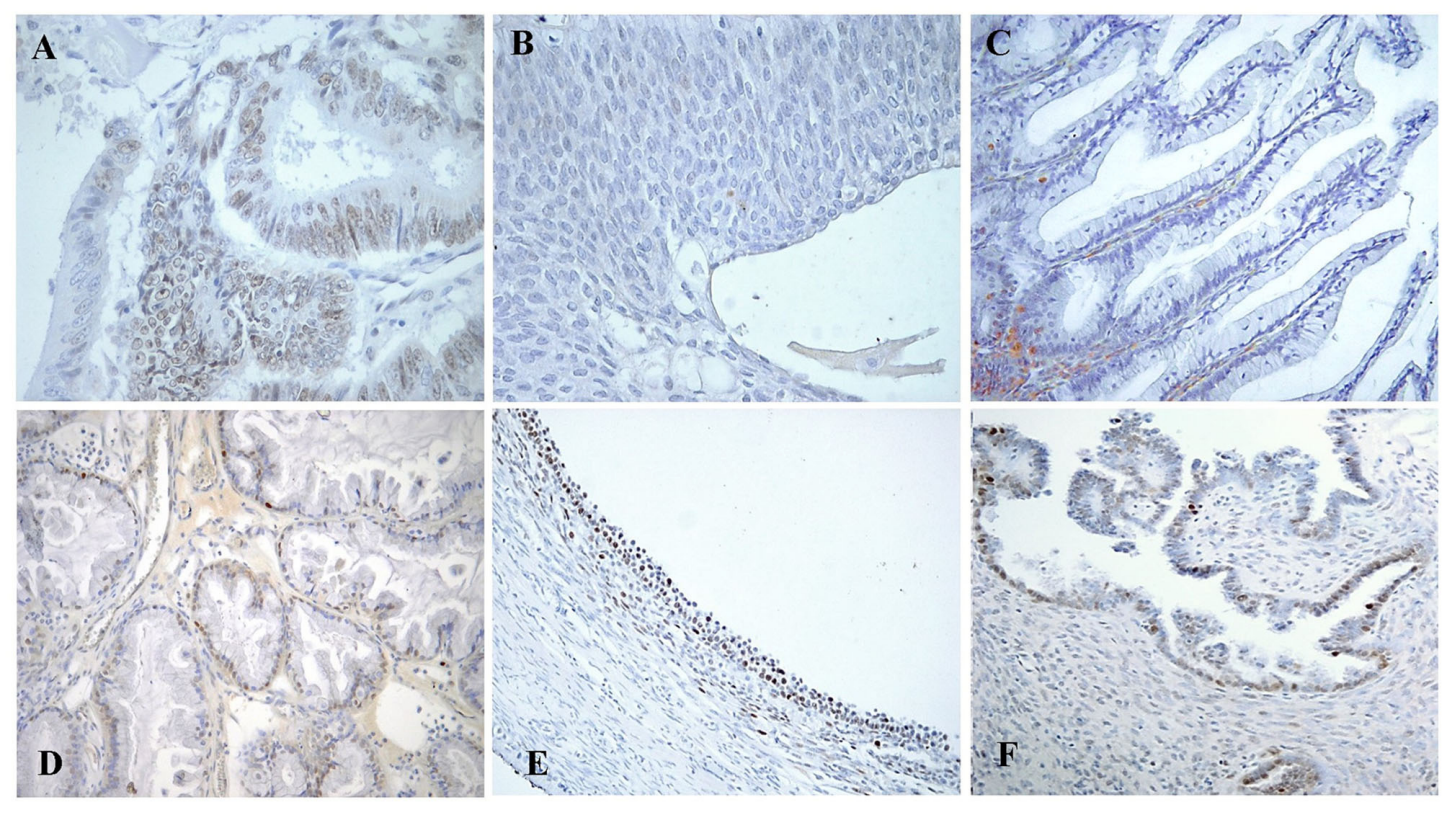
Immunohistochemical staining of Rb/p105 expression in the cells nuclei. **(A)** Colon Carcinoma as positive control for pRb (X400). **(B)** Brenner BOT as negative control for pRb (X400). **(C)** Mucinous BOT of intestinal type showing a concentration of positive nuclei at the base of the papillary projection (“Arrow”; X200). **(D)** Sero-mucinous BOT with intermediate staining of positive nuclei (X200). **(E)** Follicular cyst as an example of internal positive control for pRb (X200). **(F)** Serous BOT with lower pRb staining (X200).

Notably, the immune-reactivity for Rb/p105 was only nuclear in our series. Moreover, the nuclear Rb staining in intestinal-type mucinous BOTs tended to concentrate at the bases of the papillary projections (Figure 1).

The nuclear expression of Rb/p105 was observed in 40 (61.6%) out of the 65 patients, whereas it was not detectable in the remaining 25 cases (38.4%). The expression of nuclear Rb/p105 was more frequently detected in serous (22 cases; 84.6%) than in serous mucinous (8/19, 42.1%) and mucinous (10/20, 50%) types, and this difference was statistically significant (*P* < 0.0001). Positive staining was observed in 34 (57.6%) out of 59 stages 1 and in all 6 (100 %) stage 2/3 cases and this difference was slightly significant (*p* = 0.04). No statistically significant correlation was observed between the nuclear expression of Rb/p105, implants, and recurrences (Table 2).

### Correlation of Rb2/p130 Expression With Clinic-Pathological Parameters in BOTs

**Figure 2:**
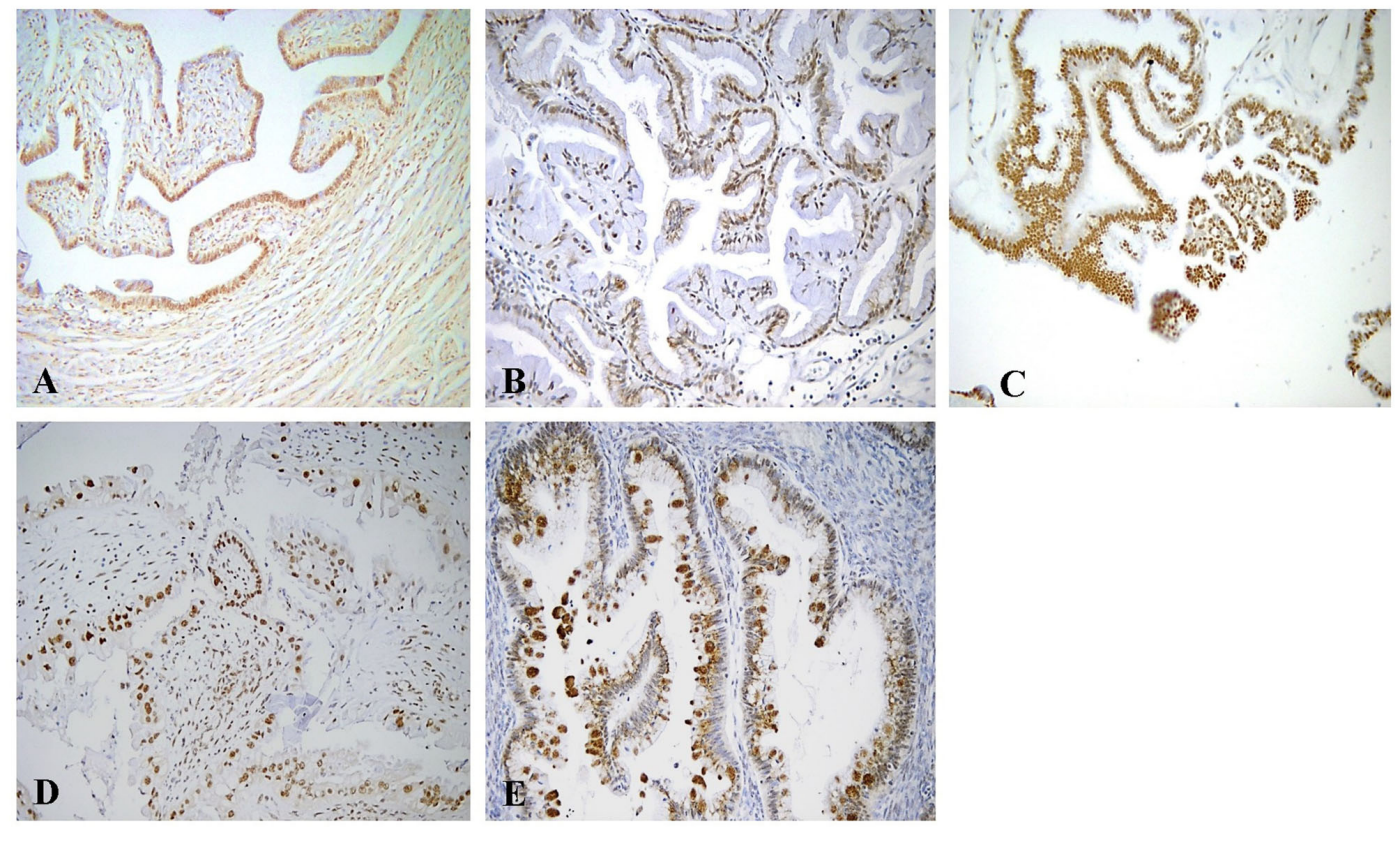
Immunohistochemical staining of Rb2/p130 expression. **(A)** Tube as internal positive control for Rb2/p130 (X100)**. (B)** Mucinous benign tumor with positive nuclei for Rb2/p130 (X200). **(C)** Serous BOT with nuclear Rb2/p130 staining (X200). **(D)** Sero-mucinous BOT with intermediate staining of positive nuclei (X200). **(E)** Mucinous BOT showing cytoplasmic Rb2/p130 staining (X200).

Nuclear Rb2/p130 expression was detected in 33 (50.8%) of the 65 cases whereas it was absent in the remaining 32 cases (40.2%) (Figure 2). The expression of nuclear Rb2/p130 was more frequent in serous (21/26, 80.8%) than in sero-mucinous (10/19, 52.6%) and intestinal (2/20, 1.0%) types and these differences were statistically significant (*P* < 0.0001). Positive staining was observed in 27 (45.7%) out of the 59 stage 1 and in all 6 (100%) stage 2/3 cases and this difference was significant (*p* = 0.03). No statistically significant correlation was observed between the nuclear expression of Rb2/p30, implants, and recurrences (Table 3A).

**Table 3A:** Nuclear distribution of Rb2/p130-negative and Rb2/p130-positive cases according to tumoral characteristics.

**Rb2/p130 Nuclear**	**Total**	**Rb2/p130 negative (0%)**	**Rb2/p130 positive (1–30%)**	**Rb2/p130 positive (>30%)**	***P***
	**N^**°**^**	**N^**°**^ (%)**	**N^**°**^ (%)**	**N^**°**^ (%)**	
**Histologic types**					<0.0001
Serous subtype	26	5 (19.2)	7 (26.9)	14 (53.9)	
Sero-Mucinous subtype	19	9 (47.4)	5 (26.3)	5 (26.3)	
Mucinous subtype	20	18 (90.0)	2(10.0)	0	
**Stage**					0.03
1	59	32 (54.3)	12 (20.3)	15 (25.4)	
2+3	6	0	2 (33.3)	4 (66.7)	
**Implants**					NS
Absent	59	31 (53.4)	12 (20.7)	15 (25.9)	
Present	6	1 (14.3)	2 (28.6)	4 (57.1)	
**Recurrences**					NS
Absent	61	30 (49.2)	12 (19.7)	19 (31.1)	
Present	4	2 (50.0)	2 (50.0)	0	

*Numbers in parentheses represent the percentage of specimens achieving that particular score. NS, not significant*.

**Figure 3:**
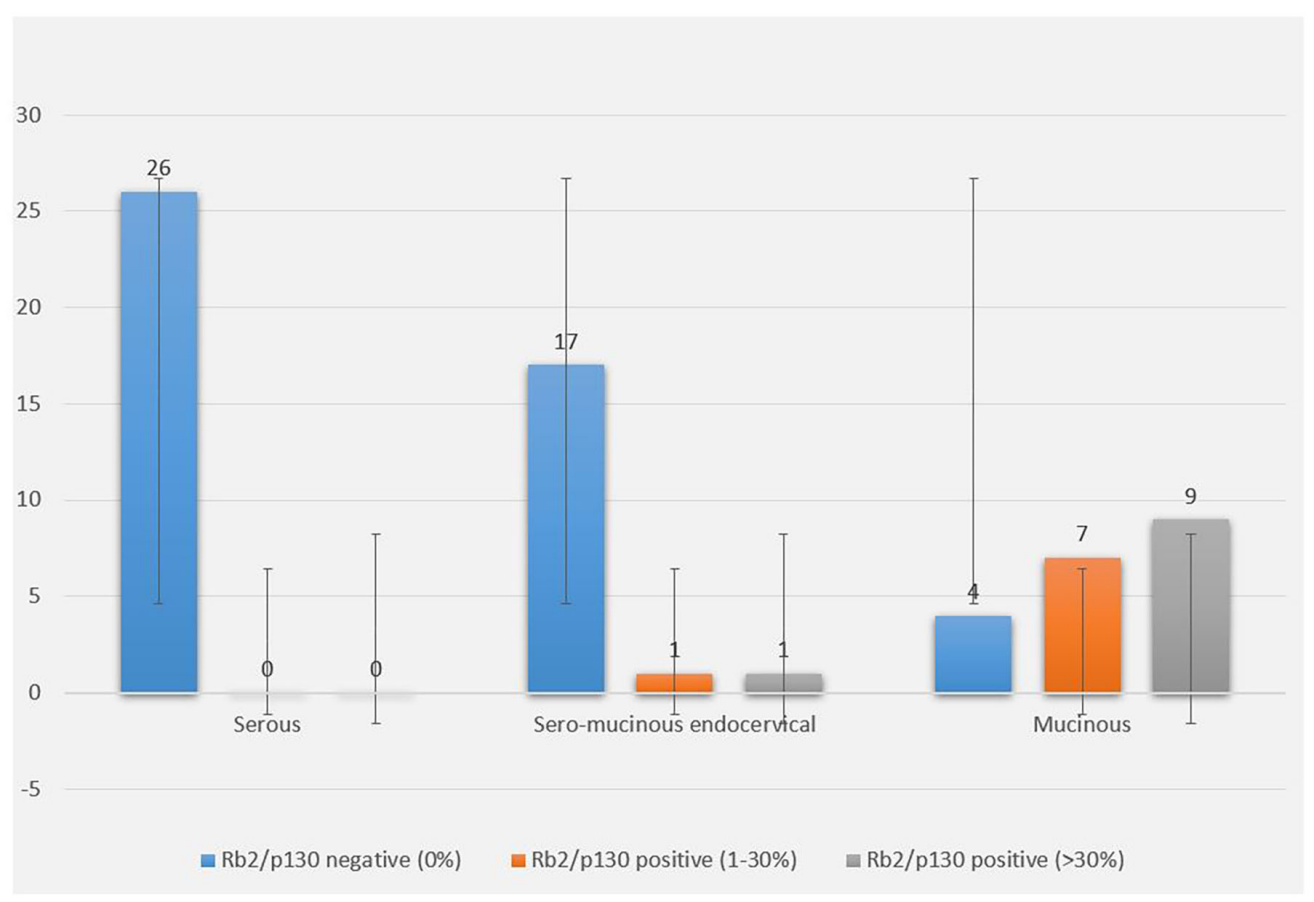
Rb2/p130 cytoplasmic expression in BOTs. The graph shows cytoplasmic distribution of Rb2/p130 expression according to histologic types, with focus on the percentage of stained neoplastic cells.

**Figure 4:**
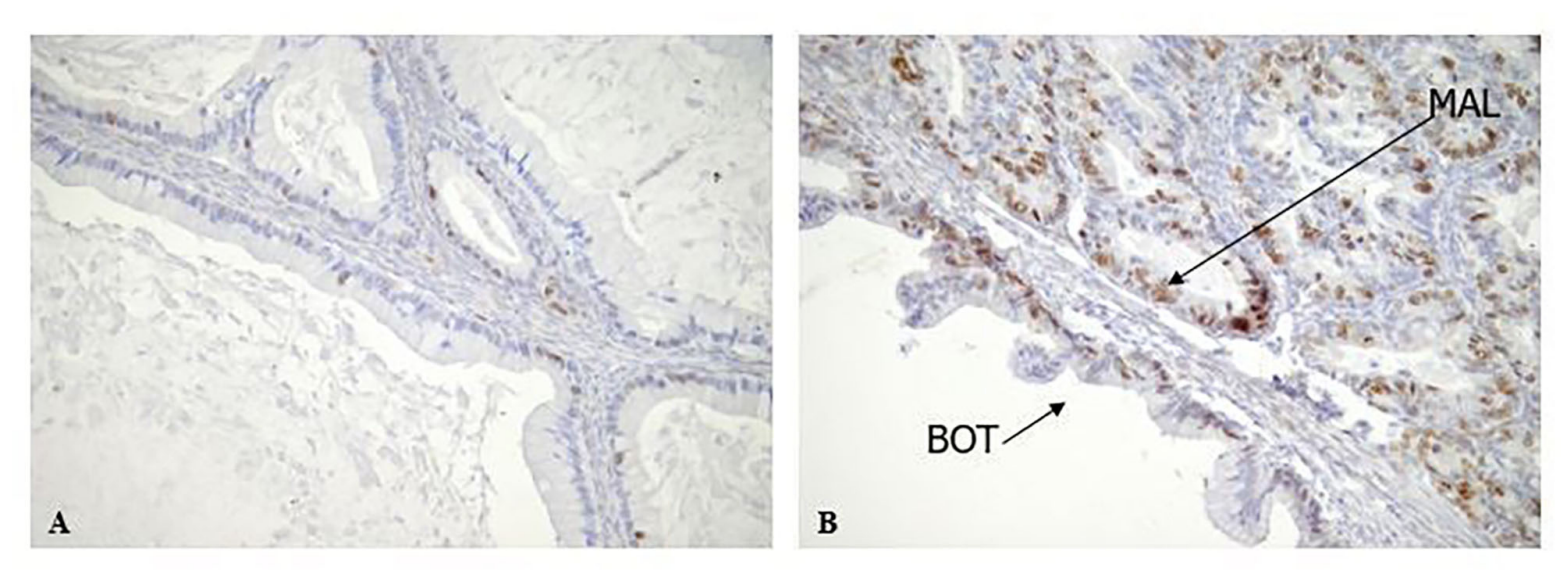
Rb2/p130 expression in Mucinous BOTs. **(A,B)** Rb2/p130 expression shows a mix of patterns, in a mucinous BOT including combination of benign, borderline and invasive carcinoma. In particular Rb2/p130 expression is nuclear in benign area (A, X200) and nuclear-cytoplasmic in borderline component (B, X200); cytoplasmic positivity has been observed in invasive carcinoma (B, X200).

The cytoplasmic expression of Rb2/p130 was detected in 18 (27.7%) cases whereas it was not evident in the remaining 47 cases (72.3%). Unlike nuclear Rb2/p130 expression, the cytoplasmic expression of Rb2/p130 was not detected in serous tumors, rarely detected in sero-mucinous (2/19, 10.6%) and frequently observed in mucinous subtypes (16/20, 80%) (Figure 3); this difference was statistically significant (*P* < 0.0001). The cytoplasmic expression of Rb2/p130 according to clinic-pathological parameters is shown in Table 3B. In Figure 4 we have shown Rb/p130 immunohistochemistry in an intestinal-type mucinous ovarian tumor composed of benign, borderline, and malignant areas. Notably, the Rb2/p130 expression moves from nuclear expression in the benign counterpart to nuclear-cytoplasmic in the BOT counterpart and cytoplasmic in the malignant counterpart (Figures 4A,B).

**Table 3B:** Cytoplasmic distribution of Rb2/p130-negative and Rb2/p130-positive cases according to histologic types.

**Rb2/p130 Cytoplasmatic**	**Total**	**Rb2/p130 negative (0%)**	**Rb2/p130 positive (1–30%)**	**Rb2/p130 positive (>30%)**	***P***
	**N^**°**^**	**N^**°**^ (%)**	**N^**°**^ (%)**	**N^**°**^ (%)**	
**Histologic types**					<0.0001
Serous subtype	26	26 (100)	0	0	
Sero-Mucinous subtype	19	17 (89.4)	1 (5.3)	1 (5.3)	
Mucinous subtype	20	4 (20.0)	7 (35.0)	9 (45.0)	

*Numbers in parentheses represent the percentage of specimens achieving that particular score*.

## Discussion

Borderline ovarian tumors (BOTs) represent about 15% to 20% of all ovarian malignancies and differ from invasive ovarian cancers by many characters (1, 2). On the one hand, they are characterized by cellular proliferation and nuclear atypia but, on the other hand, they usually do not show an infiltrative growth pattern (2). Recent knowledge supports the notion that subtypes of borderline ovarian tumors comprise distinct biologic, pathogenetic, and molecular entities, precluding a single unifying concept for BOT (2). Serous borderline tumors (SBT) share molecular and genetic alterations with low-grade serous carcinomas and can present at higher stages with peritoneal implants and/or lymph node involvement, which validates their borderline malignant potential (5). All other (non-serous) subtypes of BOT commonly present at stage I are confined to the ovary(ies) and are associated with overall survival approaching that of the general population (6, 7).

The retinoblastoma (Rb) gene family includes Rb2/p130, RB/p105, and p107 genes, which encode nuclear proteins (pRB) acting as negative regulators of cell proliferation, when in their dephosphorylated status (15, 16). Alteration of Rb family members is frequently involved in gynecological cancers (21–23).

Dong et al., in a series of 168 specimens, demonstrated high pRb expression in 41% percent of the benign, 50% of the borderline, and 71% of the malignant tumors; in this study, protein accumulation increased progressively with poorer differentiation and there was a trend for high pRb expression to be associated with an advanced stage of disease (14). Additionally, Milde-Langosch et al. demonstrated a correlation between higher pRb expression and shorter survival suggesting thus that pRb expression could play a role in early tumorigenesis, while in later stages, the tumor is independent of pRb (25).

Other authors reported significantly lower pRb levels in low malignant potential ovarian tumors (LMP) than in carcinomas and in this latter group, a reduction of pRb expression with increasing grade, advancing stage and bulk residual disease; in their study, a low pRb to Ki-67 ratio appeared as an indicator of poor survival in uni- and multivariate analysis, along with the histologic type and FIGO stage (26).

We were the first to demonstrate that loss of Rb2/p130 or its cytoplasmic expression occurs in 40% of ovarian tumors and are inversely correlated with tumor grade (24). This has been confirmed by Worley MJ et al. who evaluated the immunohistochemical Rb2/p130 expression in a series of benign, borderline (SBT), and malignant ovarian tumors (low-grade (LGSC) and high-grade (HGSC) serous carcinoma), demonstrating a significant decrease in Rb2/p130 expression during the progression from cystadenoma to SBT to LGSC. They reported no loss of expression in benign forms, whereas 10% of SBTs, 47% of LGSCs, and 16% of HGSCs had a loss of expression (27).

Differences in additional molecular markers support the idea that the serous BOTs are histologically and clinically distinct from the mucinous BOTs. For example, the expression of p21 and MDM2 differs between mucinous and serous LMP tumors (11). In other studies, a higher rate of p53 mutation was observed in mucinous relative to serous BOTs, and p21 and bcl-2 overexpression appeared specific to serous BOTs and differences among benign, borderline and malignant forms (12, 13).

In the present study, the observed differences in the expression of pRb/p105 and pRb2/p130 between serous, sero-mucinous, and mucinous BOTs supports the concept that these tumors follow different pathogenic pathways. In our series, the nuclear expression of Rb/p105 and pRb2/p130 was highly detected in serous (84.6%) compared to sero-mucinous (42.1%) and mucinous (50%) types. On the other hand, the cytoplasmic expression of Rb2/p130 was not detected in serous tumors but frequently observed in mucinous subtypes (80%). Our findings suggest that both pRb and pRb2/p130 do not play a key role in the tumor progression of serous borderline tumors since these proteins remain located in the nucleus and never showed cytoplasmic localization.

By contrast, the observed higher cytoplasmic expression of Rb2/p130 in mucinous BOTs, is suggestive of the involvement of Rb proteins in the carcinogenesis of mucinous ovarian tumors. To furtherly support our hypothesis, in Figure 4 we have shown pRb2/p130 immunohistochemistry in an intestinal-type mucinous ovarian tumor composed of benign, borderline, and malignant areas. Notably, the Rb2/p130 expression moves from nuclear expression in the benign counterpart, to nuclear-cytoplasmic in the BOT counterpart and cytoplasmic in the malignant counterpart.

Despite no statistically significant relationships between pRb immunohistochemistry and prognosis have been observed, our results may suggest that mucinous BOTs, exhibiting low nuclear and high cytoplasmic levels of Rb2/p130, may potentially be considered the BOT histotype with a higher carcinogenic risk. In fact, loss of pRb2/p130 expression has been previously reported to inversely correlate with tumor grade and to be a poor prognostic indicator in several human cancers (14, 16, 18). Moreover, its cytoplasmic localization, which implicates a loss of function, has been observed in several tumor types, including lymphoma and gastric cancer (28).

On the other hand, the normal pRb2/p130 nuclear localization, as more frequently observed in our series for serous and sero-mucinous BOT histotypes, enables its oncosuppressive function through the interaction with the E2F4 and E2F5 transcription factors.

## Conclusion

In conclusion, we have demonstrated a specific histology-related Rb proteins profile of serous, sero-mucinous, and mucinous borderline tumors.

Our findings indicate a clear role of pRb2/p130 protein in the tumor progression of intestinal-type mucinous BOTs thus suggesting a possible role of Rb proteins as prognostic factors in ovarian cancer.

Further studies on larger series are needed in order to clarify how BOTs could be stratified in different prognostic groups according to their pRb immunohistochemical profile.

## Data Availability Statement

The original contributions presented in this study are included in the article/supplementary materials, further inquiries can be directed to the corresponding author/s.

## Ethics Statement

The studies involving human participants were reviewed and approved by Fondazione Policlinico Universitario A. Gemelli IRCSS - Universita' Cattolica del Sacro Cuore, Roma, Italia. The patients/participants provided their written informed consent to participate in this study.

## Author Contributions

VM and GZ: study conceptualization and methodology design. GAn, ASa, GS, and PV: formal analysis and the original draft preparation. GAn, ASa, GAm, ASg, PV, SB, and AG: review and editing. All authors contributed to the article and approved the submitted version.

## Conflict of Interest

The authors declare that the research was conducted in the absence of any commercial or financial relationships that could be construed as a potential conflict of interest.

## Abbreviations

BOT: Borderline Ovarian Tumor
RB: Retinoblastoma
pRb: Retinoblastoma protein product or hypo-phosphorylated form
p-pRb: hyper-phosphorylated form.
